# The structure of *Plasmodium yoelii* merozoite surface protein 1_19_, antibody specificity and implications for malaria vaccine design

**DOI:** 10.1098/rsob.130091

**Published:** 2014-01-08

**Authors:** Rachel D. Curd, Berry Birdsall, Madhusudan Kadekoppala, Solabomi A. Ogun, Geoffrey Kelly, Anthony A. Holder

**Affiliations:** 1 Divisions of Parasitology, MRC National Institute for Medical Research, The Ridgeway, Mill Hill, London NW7 1AA, UK; 2 Molecular Structure, MRC National Institute for Medical Research, The Ridgeway, Mill Hill, London NW7 1AA, UK; 3 NMR Centre, MRC National Institute for Medical Research, The Ridgeway, Mill Hill, London NW7 1AA, UK

**Keywords:** malaria vaccine, MSP1 structure, plasmodium

## Abstract

Merozoite surface protein 1 (MSP1) has been identified as a target antigen for protective immune responses against asexual blood stage malaria, but effective vaccines based on MSP1 have not been developed so far. We have modified the sequence of *Plasmodium yoelii* MSP1_19_ (the C-terminal region of the molecule) and examined the ability of the variant proteins to bind protective monoclonal antibodies and to induce protection by immunization. In parallel, we examined the structure of the protein and the consequences of the amino acid changes. Naturally occurring sequence polymorphisms reduced the binding of individual protective antibodies, indicating that they contribute to immune evasion, but immunization with these variant proteins still provided protective immunity. One variant that resulted in the localized distortion of a loop close to the N-terminus of MSP1_19_ almost completely ablated protection by immunization, indicating the importance of this region of MSP1_19_ as a target for protective immunity and in vaccine development.

## 2. Introduction

Malaria is a global disease with hundreds of millions of clinical infections and at least a million deaths annually [1]. One strand of the strategy to control and finally eradicate malaria is to develop cheap and effective vaccines [2], but progress has been slow in part, because we understand poorly what is required to induce protective immunity. Individuals that survive continuous exposure to infection do eventually develop clinical immunity, suggesting that a vaccine against the asexual blood stage of the parasite is achievable.

Polymorphism in antigens such as merozoite surface protein 1 (MSP1) is an important mechanism of the malaria parasite to evade a protective immune response [3]. Variable sequences result in antigenic differences important in acquisition of immunity, for example affecting antigen processing and presentation to T cells or as epitopes for antibodies that provide protection against infection. They are an important consideration in vaccine design because efficacy against different parasite populations should be as broad as possible. MSP1_19_, the C-terminal region of MSP1 that is composed of two epidermal growth factor (EGF)-like domains, has been implicated as a target of protective immune mechanisms in a large number of studies, using molecular-, *in vitro*-, *in vivo*- and population-based methods (reviewed in [4]). Rodent parasites in laboratory mice have been used to examine immune responses to MSP1_19_; for example, immunization with recombinant *Plasmodium yoelii* MSP1_19_ may provide protection against a homologous [5,6], but not heterologous [7] parasite challenge. In natural isolates of *P. yoelii*, MSP1_19_ sequence polymorphism is extensive [8], whereas in *Plasmodium falciparum*, the most important human malaria parasite, the MSP1_19_ sequence is relatively conserved [9].

Monoclonal antibodies (mAbs) specific for *P. yoelii* MSP1_19_ that protect by passive immunization against the homologous line have been identified [10,11]. Two of these antibodies, B6 and F5, can bind to the first EGF-like domain alone, whereas a third, B10, requires both EGF domains for binding [11]. These antibodies bind to epitopes constrained by disulfide bonds [11], and to some but not all of the natural sequence variants [8]. However, the contribution of such sequence differences to immune evasion following infection is unclear.

Crystallographic and NMR-based approaches have been used to examine the three-dimensional structure of MSP1_19_ from a number of *Plasmodium* species [12–15]. Some epitopes of *P. falciparum* MSP1_19_-specific mAbs that do or do not inhibit erythrocyte invasion and parasite growth have been mapped using such structural methods or by introducing amino acid substitutions to identify the key target regions for functional antibodies [16–19]; this is particularly important because some so-called blocking antibodies compete with the binding of inhibitory antibodies, rendering them ineffective [20,21].

For vaccine development, attention has been focused on the larger 42 kDa fragment of MSP1 (MSP1_42_), but the result of phase II clinical trials of these vaccines has been disappointing [22]: despite the induction of antibody, there was no obvious clinical benefit. Therefore, we need to re-evaluate the parameters that are important in the design of candidates for vaccine development.

The antigen may be ‘engineered’ to improve desirable characteristics or remove undesirable ones [21,23], and it is important to understand the structural basis and constraints of these approaches. Amino acid differences may ablate the binding of antibodies by affecting either the local or global structure of the protein, and affect the ability of the protein to induce protection following vaccination. However, the consequence of amino acid substitutions on the structure of the protein is not predictable; the effects may be localized if the residue is on the surface, or profound, particularly if the substitution is a radical change in the side chain. Such changes may also affect B-cell epitopes, and antigen processing and presentation to T cells [24]. There have been no experimental studies to explore the relationship between amino acid sequence, protein three-dimensional structure and antigenic polymorphism in protection against malaria.

To examine some of these aspects experimentally, we used the *P. yoelii* model. We produced recombinant MSP1_19_ protein with single amino acid sequence differences either at positions where variation is found naturally [8] or within a conserved region. We examined the effects of these changes on the binding of protective mAbs and on the three-dimensional structure of the protein. Then, we examined the immunogenicity of the modified proteins and their ability to provide protection against parasite challenge.

## 3. Material and methods

### 3.1. Ethical statement

All animal work protocols were reviewed and approved by the Ethical Review Panel of the MRC-NIMR, and approved and licensed by the UK Home Office as governed by law under the Animals (Scientific Procedures) Act 1986 (project licence no. 80/1832). The experimental procedures were designed to minimize the extent and duration of any harm, and included predefined clinical and parasitological endpoints to avoid unnecessary suffering.

### 3.2. Preparation of wild-type and variant MSP1_19_ proteins

Production in *Escherichia coli* and purification of a *P. yoelii* YM WT GST fusion protein (GST-MSP1_19_) has been described previously [6]. Four variant proteins with single amino acid changes—R12L, K16E, N17H and E28K (residues numbered according to the N-terminus of MSP1_19_)—were produced following site-directed mutagenesis. The wild-type (WT) and variant proteins with an N-terminal hexa-His tag were also produced in *Pichia pastoris* using a synthetic gene with the *N*-glycosylation site removed, and inserted into the pPICK9K vector (Invitrogen), with transformation and selection of expressing cells.

### 3.3. Antigenic analysis of wild-type and variant GST-MSP1_19_ proteins

Binding of the mAbs B6, B10 and F5 [11] to GST-MSP1_19_ variants was analysed by Western blotting, ELISA and surface plasmon resonance (SPR). For Western blotting, the proteins were electrophoresed on NuPAGE Bis–Tris 12% polyacrylamide gels under non-reducing conditions and transferred onto nitrocellulose. These conditions were used because cystine reduction or reduction and alkylation either severely reduces or abolishes reactivity with the mAbs [11]. Following blocking with 5% (w/v) skimmed milk in PBS, blots were incubated with mAbs (B6, 2 μg ml^−1^; B10, 2 μg ml^−1^; F5, 10 μg ml^−1^), and binding was visualized using the ECL kit (Amersham). For ELISA analysis of mAb binding, 96-well plates (Nunc Maxisorp) were coated with anti-GST antibody followed by 1 μg ml^−1^ of WT or variant GST-MSP1_19_ and PBS- and GST-alone controls. Doubling dilutions of primary antibody were added, followed by goat anti-mouse IgG-HRP and detection with *O*-phenylenediamine dihydrochloride at 490 nm. Surface plasmon resonance measurements were made on a BIAcore 2000 instrument with anti-GST antibody bound to the surface of a CM5 sensor chip and GST-MSP1_19_ proteins bound at 10 µg ml^−1^. mAb binding assays were performed at a constant flow rate of 5 μl min^−1^ at 25°C, using B6 (1.29 mg ml^−1^), B10 (1.18 mg ml^−1^) and F5 (1.18 mg ml^−1^), recording the steady-state binding (within 2 min) in triplicate repeated binding assays.

### 3.4. Immunization studies with wild-type and variant GST-MSP1_19_ proteins

Groups of six eight-week-old BALB/c mice were immunized intraperitoneally (i.p.) with 10 µg protein in Freund's complete adjuvant, and boosted by two further injections of 40 µg of protein in Freund's incomplete adjuvant 21 and 42 days later. Serum samples were taken and pooled for each group 14 days after the final immunization, and the titre of MSP1_19_-specific antibodies was measured by ELISA, coating the wells with 100 μl of 1 μg ml^−1^ WT MSP1_19_ produced in *P. pastoris* and detecting bound antibody as described above. Fifteen days after the last immunization, mice were challenged by intravenous (i.v.) injection of 5 × 10^3^ *P. yoelii* YM-parasitized erythrocytes. The parasitaemia was followed by microscopy of blood films stained with Giemsa's reagent, daily from day 3 for at least 21 days and until the mice had cleared the parasites. The geometrical mean of parasitaemia was calculated from the percentage of parasite-infected erythrocytes in individual mice. Peak parasitaemia differences (*n* = 6 for each group) were analysed by one-way ANOVA and Tukey's honestly significance difference test was performed for *post hoc* analysis. Differences in parasitaemia during the course of the infection (25 days) were analysed by Wilcoxon matched-pairs signed-rank test. A *p*-value of less than 0.05 was considered significant. Differences in survival between groups were analysed by the log-rank test. The statistical analysis was performed with R package (v. 2.15.1).

### 3.5. NMR assignments and structure calculations

NMR spectra were recorded at 25°C or 35°C on Varian Inova (600 and 800 MHz) and Bruker Avance (600 and 700 MHz) spectrometers equipped with triple resonance z-gradient probes. All spectrometers except for the Varian 600 MHz were equipped with cryogenic probes. Water suppression was carried out using the WATERGATE sequence [25]. ^1^H{^15^N}-HSQC spectra were acquired for ^15^N-labelled WT MSP1_19_ and all variants. For the WT and the E28K variant, three-dimensional NMR experiments (HNCO, HNCACB and CBCA(CO)NH) were used for making the sequential assignments. Aliphatic and aromatic side chain assignments were derived from three-dimensional HCCH-TOCSY, HCCCONH and ^13^C-edited NOESY-HSQC spectra recorded separately for the aliphatic and aromatic regions. For the WT MSP1_19_, chemical shift assignments were obtained for more than 97% of the ^1^H, ^13^C and ^15^N atoms of the protein backbone, and for more than 78% of the side chain atoms; similar levels were achieved for the E28K MSP1_19_. The first 10 residues (His tag and linker region) are not included. All spectra were processed with NMRPipe software, visualized with NMRDraw [26] and analysed using XEASY or Sparky software (T. D. Goddard and D. F. Kneller, Sparky 3, UCSF, USA).

Amide proton exchange rates were measured in a series of SOFAST ^1^H{^15^N}-HMQC spectra recorded every 5 min in a pseudo-three-dimensional experiment. To evaluate quantitatively the extent of protection of the amide protons, the H/D exchange rates were measured from the time course of the NH peak intensities over 2–3 h.

Distance restraints for calculating the families of structures for WT and E28K proteins were obtained from nuclear Overhauser effects (NOEs) in four spectra: an ^15^N-edited NOESY-HSQC, separate ^13^C-edited NOESY-HSQC spectra for the aliphatic and aromatic regions, and a two-dimensional ^1^H NOESY acquired for a sample in 99% ^2^H_2_O; all NOESY experiments used a mixing time of 100 ms. Peaks from the NOESY spectra were picked manually using Sparky. The spectra were transferred to XEASY and the peak volumes determined by integration [27]. The NOE peak lists for the four spectra together with the chemical shift lists for all assigned nuclei and a list of dihedral angle restraints from TALOS [28] were submitted to ARIA 1.2 and 2.2 software packages [28–32] for structure calculation using default settings*.* NOEs were assigned via an iterative procedure using ARIA, and violated restraints were checked manually in Sparky. Potential hydrogen bonds in preliminary structures were calculated using MOLMOL [33]. H bonds calculated to be in eight or more of the 10 structures, and confirmed by the persistence of an NH crosspeak in the ^2^H_2_O exchanged ^1^H{^15^N}-HSQC spectrum, were included in subsequent ARIA calculations. The structures were also examined to identify the disulfide bonds [34], which were added to the ARIA structure calculations initially as restraints and then as covalent bonds. For the final ARIA runs in iteration 8, the number of structures was increased to 100, and the 20 best energy structures were used in the water refinement. The quality of the final structures was assessed using PROCHECK NMR [35], and MOLMOL was used to calculate the surface electrostatic potential. Structure visualization, analysis and comparisons were carried out using InsightII (Accelrys Software Inc.).

The total number of NOE-derived distance restraints used in the final calculations was 3391 for the WT and 2927 for the E28K variant. A breakdown of the NOEs is given in the electronic supplementary material, table S1. The ^1^H , ^15^N and ^13^C chemical shifts have been deposited in the BioMagResbank database (http://www.bmrb.wisc.edu) under the accession numbers 19233 and 19234. The structural data and experimental restraints used in calculations have been submitted to the Protein Data Bank with PDB ID codes 2mgp and 2mgr.

## 4. Results

### 4.1. The effects of single amino acid substitutions on mAb binding to MSP1_19_

**Figure 1.**
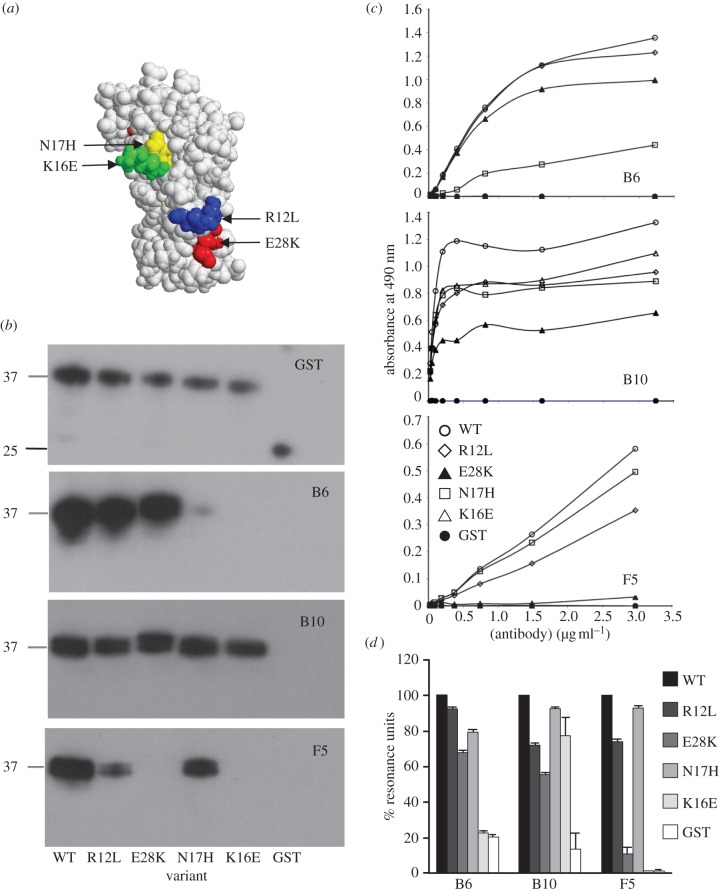
Single amino acid changes reduce the binding of protective monoclonal antibodies to *P. yoelii* MSP1_19_. (*a*) The location and nature of the four amino acid substitutions examined in this study. Antibody binding was examined by (*b*) Western blotting, (*c*) ELISA and (*d*) surface plasmon resonance. The mAbs B6, B10 and F5 were used to probe either GST or GST-MSP1_19_ with the WT sequence or the four variants R12L, E28K, N17H and K16E. (*b*) Anti-GST antiserum was used as a control and the location of GST (25 kDa) and the GST-MSP1_19_ fusion proteins (37 kDa) is indicated. (*c*) ELISA plates coated with each antigen were probed with increasing dilutions of the individual mAbs. (*d*) Chips were coated with the different proteins and probed with the individual antibodies.

We produced recombinant proteins containing either single natural polymorphisms (R12L, K16E and N17H) or a substitution of a conserved residue, changing the charge (E28K; figure 1*a*). In order to test whether the amino acids changed in the MSP1_19_ variants affected the binding of the protective B6, F5 and B10 mAbs [11], Western blotting, ELISA and SPR analyses were carried out using the WT and variant proteins (figure 1). For the F5 antibody, K16E and E28K abolished, R12L reduced and N17H had little effect on binding in all assays. For B6, K16E largely abolished, N17H reduced and R12L had no effect on binding. For the antibody B10, R12L and E28K reduced binding in the ELISA and SPR assays, but the differences were less pronounced on the Western blot. Overall, each variant gave a different pattern of reactivity, but no single amino acid change resulted in the loss of binding of all three mAbs, indicating that their contribution to the evasion of antibody-mediated protection is likely to be cumulative.

### 4.2. The effects of amino acid substitutions on the structure of MSP1_19_

**Figure 2.**
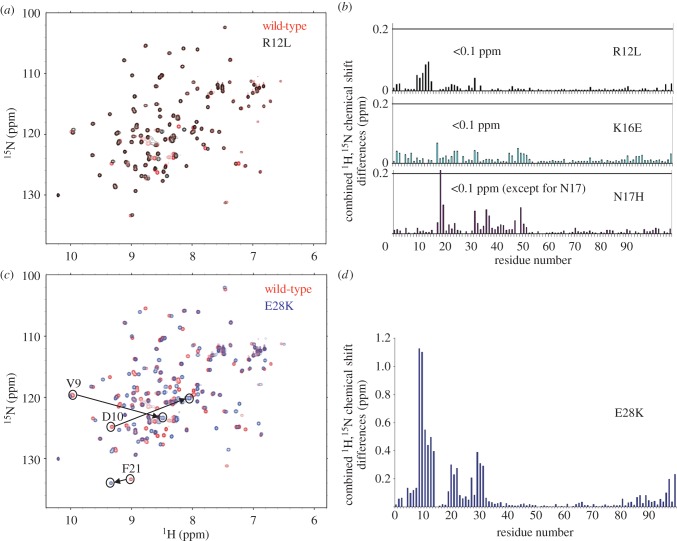
The effects of single amino acid substitutions on the structure of MSP1_19._ (*a*) Comparison of the ^1^H{^15^N}-HSQC spectra for WT and R12L MSP1_19_. (*b*) Combined chemical shift differences (|Δ*δ*(15N)|/5 + |Δ*δ*(1H)|)/2) between WT and R12L, K16E and N17H proteins, respectively. (*c*) Comparison of the ^1^H{^15^N}-HSQC spectra for WT and E28K MSP1_19_; the shifts for three residues V9, D10 and F21 are highlighted. (*d*) Combined chemical shift differences between WT and E28K. The resonance assignments for the WT protein are provided in the electronic supplementary material, figure S1; the comparison of WT with K16E and N17H proteins is shown in the electronic supplementary material, figure S2.

To understand the basis of the reduced binding of the mAbs, the structural consequences of these amino acid substitutions were monitored using NMR spectroscopy. Comparison of the ^1^H{^15^N}-HSQC spectra of the variants with the WT MSP1_19_ spectrum (see electronic supplementary material, figure S1) and quantification of chemical shift changes identified regions where changes in the structure had occurred. For the R12L, K16E and N17H variants, the ^1^H{^15^N}-HSQC spectra were very similar to the WT spectrum (comparison of WT and R12L shown in figure 2*a*; comparison of WT with K16E and with N17H shown in the electronic supplementary material, figure S2). Figure 2*b* shows histograms of the weighted ^1^H/^15^N chemical shift difference for each residue of these three variants and WT MSP1_19_. The chemical shift changes were small, associated with residues at or near the substitution, and the spectra of the variants could be assigned by comparison with the WT spectrum (see below). However, for the E28K variant, the two spectra were very different and a large number of NH peaks had shifted (figure 2*c*), necessitating a full, independent assignment for this variant. Comparison of the E28K and WT chemical shifts (figure 2*d*) showed that 18 of the 48 residues in the first EGF domain had amide shifts of greater than 0.1 ppm, and two of them were greater than 1.0 ppm. By contrast, the amides of only the three terminal residues of the second EGF domain shifted by more than 0.1 ppm. Clearly, the E28K variant is having a larger effect on the first rather than on the second EGF domain; interestingly, the largest shift changes were for residues 9–14 and not at the site of the changed residue, position 28.

The direction and magnitude of the chemical shift changes seen for residues 9 and 10 suggested that H bond interactions may have been lost in the E28K variant. ^2^H_2_O exchange experiments to determine the rates of exchange of individual amide residues were performed (see electronic supplementary material, figure S3); rates for residues in the second domain were almost identical in the WT and E28K proteins (although two residues could not be quantified owing to overlap). By contrast, 14 residues in the first domain exhibited reduced protection from exchange in the variant, and residues 7–10 protected in the WT protein showed no protection in the variant. These data, together with the chemical shift changes, support the view that residues 9 and 10 are not H-bonded in E28K.

### 4.3. Comparison of the three-dimensional structure of wild-type and E28K MSP1_19_

**Figure 3.**
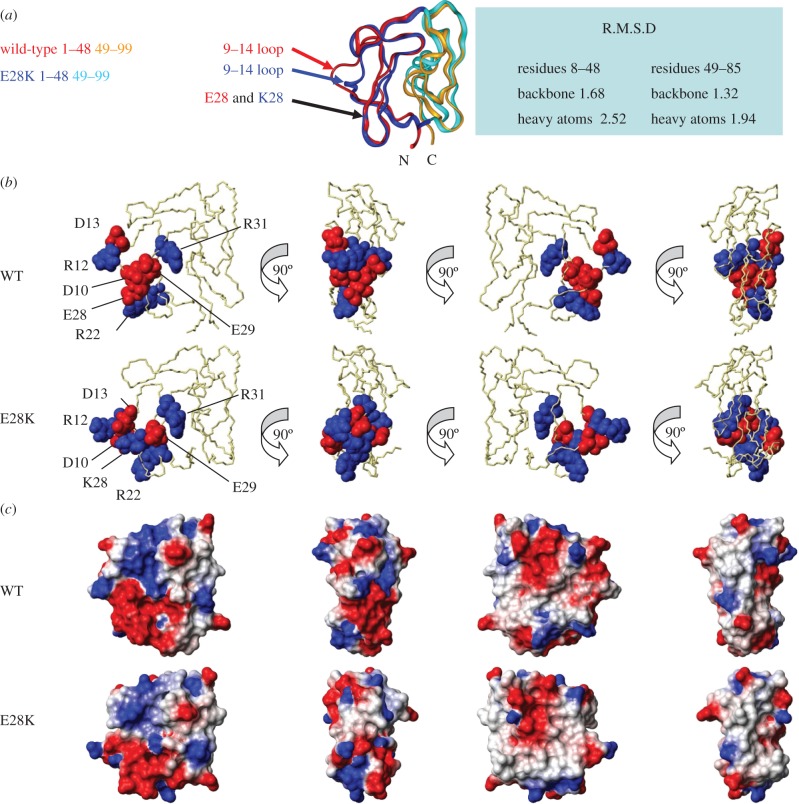
The structure of WT and E28K MSP1_19._ (*a*) Superimposition of WT and E28K MSP1_19_ in a ribbon representation; the first EGF domain (residues 1–48) is coloured red and blue, and the second EGF domain (residues 49–99) is coloured orange and cyan, respectively. (*b*) Ribbon representation with charged residues that have altered position highlighted, and (*c*) electrostatic surface potential with selected charged residues indicated. (*b*,*c*) Four views of the molecules with a 90° rotation between each view are presented; Arg and Lys residues are depicted in blue and Asp and Glu residues are depicted in red. Data that support these structures include D_2_O exchange rates (see electronic supplementary material, figure S3); the 20 lowest-energy structures for each protein are presented in the electronic supplementary material, figure S4.

In the light of the substantial chemical shift differences between WT and E28K MSP1_19_, complete structure determinations of both were undertaken to allow full comparison. The numbers of NOE distance restraints used in the final iteration of the ARIA structure calculations are shown in the electronic supplementary material, table S1; the Ramachandran plot quality is typical of that found for other EGF structures [36]. Electronic supplementary material, figure S4 shows the backbone traces of the 20 lowest-energy structures for WT and E28K MSP1_19_; the traces for both families showed good convergence especially for the interior regions, although the ends of some loops (23–26, 40–42 and 73–75) exhibit more variability. Figure 3*a* shows the lowest-energy structures of the two proteins superimposed using residues 8–85 (the less well-defined end residues are not useful for super-positioning) to identify differences between them. The major difference between the two proteins is the conformation of the residue 9–14 loop. In the orientation shown, the WT loop appears in the plane of the paper, whereas, in the E28K variant, it appears perpendicular to the page. A manual analysis of the NOE connectivities observed for this loop region indicated a subtle change in the local packing. Changes in NOE patterns include the observation of strong NOEs from T11 to W30 in the WT protein. These are absent in the E28K variant, which instead exhibits strong connectivities between T11 and C20/F21. The backbone amide proton exchange data (see electronic supplementary material, figure S3) also support a change in packing interactions. There are also some differences in the conformation of the last residues, 89–99, where the two EGF domains are in contact. RMSD values calculated for the superimposition indicate that the structures are broadly similar, with the main changes in the first domain (figure 3*a*).

Antibody binding to this protein will be sensitive to changes in the conformation of the side chains of the surface residues. Although surface residue side chains are not as well defined as buried residues in these families of structures, the surface side chains do not have complete freedom and are concentrated in a narrow region of conformational space (figure 3*b*). Therefore, it is possible to say that the side chains of certain surface residues (for example the charged residues, D10, R12 and D13) have changed their position in response to the change at residue 28. In the WT protein, the side chain of E28 is less than 5 Å from the D10 side chain, but very far from the R12 and D13 side chains. However, in the variant, the K28 side chain is near to the side chain of both D10 and D13. R12 and D13 appear to have swapped places, with D13 moving towards K28 and R12 moving away. The electrostatic surface potential for the WT and E28K proteins was mapped onto the two lowest-energy structures using MOLMOL, revealing two distinct clusters of positively and negatively charged residues on the two proteins (figure 3*c*).

### 4.4. Effect of single amino acid substitutions on the ability of MSP1_19_ to protect against parasite challenge *in vivo*

**Figure 4.**
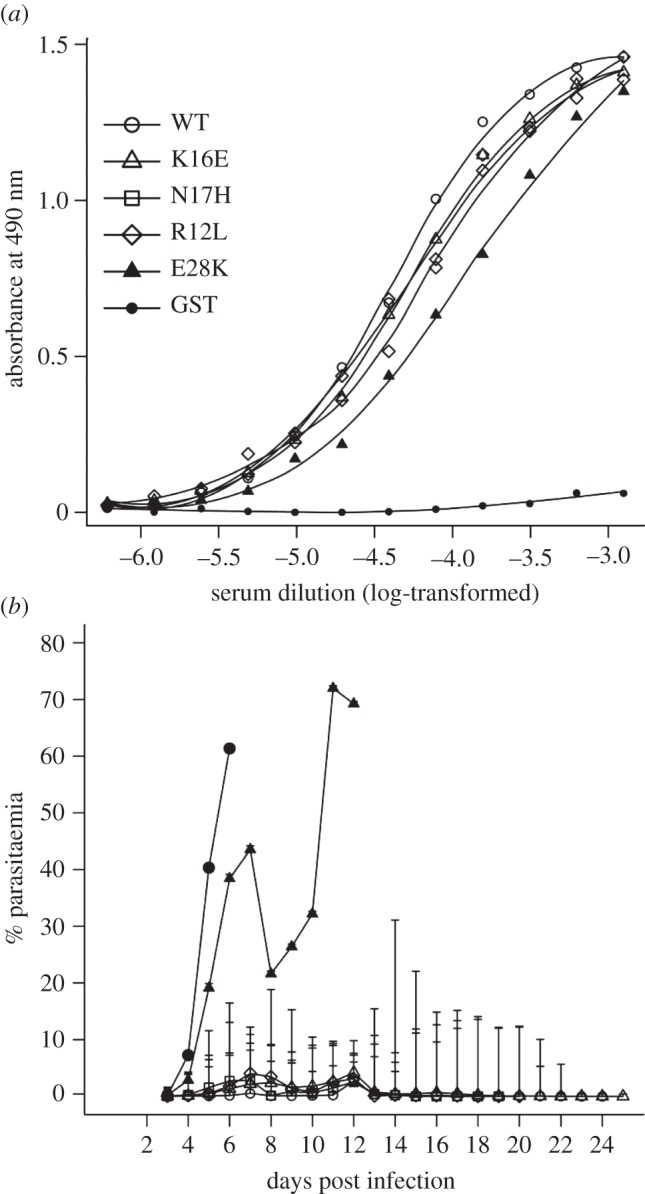
Immunogenicity of the proteins and their ability to provide protective immunity against a parasite challenge. (*a*) ELISA analysis of induced antibodies using WT protein. GST or individual GST-fusion proteins were used to immunize groups of mice, then serum samples for each group were pooled and the antibodies elicited were titrated against WT MSP1_19_ produced in *P. pastoris*. (*b*) Effect of single amino acid substitutions on the ability of MSP1_19_ to protect against parasite challenge *in vivo.* Individual groups of mice were immunized with the recombinant proteins shown on the left-hand side of panel (*a*) and then challenged with *P. yoelii*-infected erythrocytes. The resultant geometric mean parasitaemia is plotted against the days after challenge. Some mice were sacrificed during the experiment if the parasitaemia exceeded 70% or if they appeared to be in poor health: in the GST group, six mice on day 6; in the E28K group, four mice on day 8 and two on day 12; in the WT group, one on day 8; in the R12L group, one on day 9 and one on day 18; in the K16E group, one on day 9; and in the N17H group, three on day 8. The drop in parasitaemia on day 8 for the E28K group is explained by the fact that only the two animals with the lowest parasite burden survived beyond day 7. Analysis of the survival data is presented in the electronic supplementary material, figure S5.

Immunization studies were designed to look at the effect of the sequence changes on the ability of MSP1_19_ to provide protection against challenge infection with the lethal *P. yoelii* YM parasite by vaccination. Following immunization and prior to challenge, a serum sample was taken from each mouse and pooled for each group, and the antibody titre against WT MSP1_19_ was measured (figure 4*a*). Mice immunized with each of the variants had produced antibodies binding to MSP1_19_, and overall there was little difference in the antibody titres, indicating that the structural differences between the variants had no major effect on the induction of polyclonal antibodies reactive with the WT protein. Following challenge, the parasitaemia was followed daily from day 3 on Giemsa-stained blood films (figure 4*b*). None of the mice immunized with GST or E28K was protected. In the GST-alone group, parasites were patent on day 4, and by day 6 all the animals had a high parasitaemia and were sacrificed. In the E28K group, parasitaemia rose rapidly to day 7 and all mice had to be sacrificed due to poor clinical condition or high parasitaemia. By contrast, the overall patterns of parasitaemia for the WT, R12L, K16E and N17H groups were very similar, with low parasitaemia and final resolution of the infection, although some mice died throughout the experiment. A significant difference in peak parasitaemia was observed between WT and E28K groups (ANOVA, *p* = 0.003), and WT and GST groups (ANOVA, *p* < 0.0001). The geometrical means of parasitaemia during the course of infection also differed significantly for E28K (Wilcoxon matched-pairs signed-rank test, two-tailed *p* < 0.005) and for K16E (two-tailed *p* < 0.0001) groups from WT. Although the K16E group mean parasitaemia was higher than that of the WT group, the mice resolved parasitaemia, revealing significant protection from infection. The Kaplan–Meier estimates of survival probability are shown in the electronic supplementary material, figure S5. In comparison with the mice that received WT MSP1_19_, the mice immunized with the E28K variant showed decreased survival rates following parasite challenge (*p* = 2.48 × 10^−05^). On the other hand, the mice that received the R12L, K16E and N17H variants did not exhibit a significant difference in survival rate when compared with the WT protein-immunized mice, with *p*-values of 0.634, 0.998 and 0.317, respectively. Overall, the results indicate that proteins containing individual natural variants still induced protective antibodies. The variant with the greatest effect on protein structure still induced antibodies reactive with the WT protein, but these antibodies were not protective.

## 5. Discussion

MSP1 remains a candidate for inclusion in a malaria vaccine. Much attention has been focused on the C-terminal region of this molecule, particularly MSP1_19_, the last approximately 100 amino acids preceding the GPI addition site [4]. This region is the target of antibodies that inhibit parasite growth by a variety of mechanisms [37]. The fine specificity of the antibody is crucial, and the binding sites of antibodies with different properties have been defined using a variety of techniques (reviewed in [4]). For example, amino acid substitutions in *P. falciparum* MSP1_19_ have been used to map the binding of ‘inhibitory’, ‘neutral’ and ‘blocking’ antibodies [4,19]. Few studies have been carried out to determine the protective capacity of antibodies induced by immunization with such antigens [38–40]. Passive immunization with *P. yoelii* MSP1_19_-mAbs can suppress a blood stage infection [10–11], and these antibodies react with some, but not all recombinant MSP1_19_ of other *P. yoelii* lines, suggesting that the natural polymorphisms they contain represent antigenic diversity important in strain-specific immunity [8]. Immunization with MSP1_19_ provides protection against homologous blood stage challenge. MSP1_19_ sequence is conserved across species, but a number of amino acid substitutions are either species-specific or polymorphic within a single species [8,12].

Here, we have made several variant MSP1_19_ proteins, and examined the consequences of these changes on the structure of the protein, its ability to bind to different antibodies and its ability to induce protective immune responses to infection. We produced four variant proteins. Two (K16E and N17H) are natural variants in *P. yoelii*, and both these changes correlate with lack of binding of the mAbs B6 and F5. Across *Plasmodium* spp. K16 is not conserved, but residue 17 is almost always Asn. A third variant (R12L) is at a residue that is largely conserved within *P. yoelii* but may differ in different *Plasmodium* species [8]. These changes produced only very local structural perturbations; nevertheless, they abolished binding of one or more specific mAbs. The modified proteins induced antibodies that react with the WT protein, and immunized animals were partially protected against parasite challenge by a mechanism that is probably antibody-mediated [6]. This suggests that these epitopes, although the target of protective mAbs, are not individually sufficient as targets for protective immunity, and therefore it is likely that immune selection has driven cumulative polymorphisms in which incremental structural changes have occurred to evade protective antibody.

The final variant (E28K) is at a residue invariant across all species of malaria parasite. This Glu residue sits on the surface of the molecule, but changing it had profound consequences for a small region of the three-dimensional structure of the protein. Substitution of Glu by the positively charged Lys had profound effects on its ability to provide protection in immunization experiments. When used to immunize mice no protection was provided against parasite challenge, even though antibodies were produced that reacted at a similar but somewhat reduced level with the WT protein, and consistent with the perturbation being restricted to a small part of the molecule. The induction of these antibodies rules out the loss of protection due to this protein being degraded more rapidly following immunization.

To understand the extent of the perturbation, we solved the solution structure of both the WT and E28K variant proteins. The substitution had resulted in a reorganization of the charged residues within a region of the first EGF domain to accommodate the change, and other regions of the molecule remained unperturbed. These results identify a small region of the first EGF domain of MSP1_19_ for which the structural integrity is very important in the induction of a protective immune response. This region is highlighted in the sequence alignments and three-dimensional structure comparisons for MSP1_19_ from a number of *Plasmodium* species shown in the electronic supplementary material, figure S6.

It is unclear whether the E28K substitution in the parasite MSP1 would reduce viability. To test this possibility, the specific change could be introduced into the parasite by transfection and the effect on growth assessed. However, it seems unlikely that the change would be detrimental, because the entire MSP1_19_ sequence can be replaced by the two EGF domains of MSP8 [41].

This study highlights the importance of relating the structural consequences of changes to an antigen that might result from ‘engineering’ to the functional outcome. ‘Antigen engineering’ seeks to improve immunogenicity by focusing the immune response on the ‘right’ epitopes, a process that has been called ‘immunofocusing’ [23]. In the case of MSP1_19_, different antibody specificities have been defined, including a class that blocks the binding of neutralizing antibody [21]. Changes in primary sequence may also result in changes in antigen processing and presentation to T cells by dendritic cells or the presence of T-cell epitopes, both of which may either suppress or enhance the immunogenicity of the protein [24,42]. However, the structural consequence of engineered amino acid changes is poorly understood. With respect to vaccines for human malaria that contain MSP1, some progress has been made in the identification of amino acid substitutions that can be made with a view to improving the immunogenicity of the protein. However, the current inability to easily carry out *in vivo* challenge studies with *P. falciparum* restricts progress. ‘Falciparumized’ rodent parasites in which the MSP1 EGF domains have been replaced by the equivalent region from other malaria species [43,44] have been used [45], but parasites that contain at least the full-length *P. falciparum* MSP1_19_ [46] and longer chimeric or full-length MSP1 are required to be able to easily test the outcome of modifications to this molecule.

We conclude that individual amino acid changes that occur naturally in MSP1_19_ can abolish the binding of individual protective mAbs, an observation made previously with other malarial antigens, such as apical membrane antigen 1 [47]. However, such amino acid changes may have a small localized effect on the structure of the protein and do not substantially reduce the protection provided by immunization with the protein. On the other hand, at least one amino acid substitution did not affect the binding of all of the protective mAbs, but had a profound effect on the structure of part of the first EGF domain. Although the protein-induced antibodies reacted with the WT protein, they were unable to provide protection against parasite challenge. This result identifies an important structural target for the development of protective antibodies, and with a fuller understanding of the importance of structural differences for immunogenicity and protection it should be possible to design new and more effective antigens for inclusion in malaria vaccines. For example, antigens that induce a strong antibody response to the first loop of the first EGF domain would be of particular interest. Furthermore, in a complementary approach, the design of antigens to quantify the level of antibody response to this region of the molecule is possible and could facilitate studies to obtain *in vitro* surrogate markers of protection.

## Supplementary Material

Py MSP1 supplementary table and figures

## References

[1] Murray CJ 2012 Global malaria mortality between 1980 and 2010: a systematic analysis. Lancet 379, 413–431. (doi:10.1016/S0140-6736(12)60034-8) 22305225 10.1016/S0140-6736(12)60034-8

[2] Vaccines malERA Consultative Group. 2011 A research agenda for malaria eradication: vaccines. PLoS Med. 8, e1000398 (doi:10.1371/journal.pmed.1000398) 21311586 10.1371/journal.pmed.1000398 PMC3026701

[3] Thera MA 2011 A field trial to assess a blood-stage malaria vaccine. N. Engl. J. Med. 365, 1004–1013. (doi:10.1056/NEJMoa1008115) 21916638 10.1056/NEJMoa1008115 PMC3242358

[4] Holder AA 2009 The carboxy-terminus of merozoite surface protein 1: structure, specific antibodies and immunity to malaria. Parasitology 136, 1445–1456. (doi:10.1017/S0031182009990515) 19627632 10.1017/S0031182009990515

[5] Daly TM Long CA 1993 A recombinant 15-kilodalton carboxyl-terminal fragment of *Plasmodium yoelii yoelii* 17XL merozoite surface protein 1 induces a protective immune response in mice. Infect. Immun. 61, 2462–2467. 8363656 10.1128/iai.61.6.2462-2467.1993 PMC280869

[6] Ling IT Ogun SA Holder AA 1994 Immunization against malaria with a recombinant protein. Parasite Immunol. 16, 63–67. (doi:10.1111/j.1365-3024.1994.tb00324.x) 8015856 10.1111/j.1365-3024.1994.tb00324.x

[7] Renia L Ling IT Marussig M Miltgen F Holder AA Mazier D 1997 Immunization with a recombinant C-terminal fragment of *Plasmodium yoelii* merozoite surface protein 1 protects mice against homologous but not heterologous *P. yoelii* sporozoite challenge. Infect. Immun. 65, 4419–4423. 9353014 10.1128/iai.65.11.4419-4423.1997 PMC175635

[8] Benjamin PA 1999 Antigenic and sequence diversity at the C-terminus of the merozoite surface protein-1 from rodent malaria isolates, and the binding of protective monoclonal antibodies. Mol. Biochem. Parasitol. 104, 147–156. (doi:10.1016/S0166-6851(99)00142-5) 10593171 10.1016/s0166-6851(99)00142-5

[9] Miller LH Roberts T Shahabuddin M McCutchan TF 1993 Analysis of sequence diversity in the *Plasmodium falciparum* merozoite surface protein-1 (MSP-1). Mol. Biochem. Parasitol. 59, 1–14. (doi:10.1016/0166-6851(93)90002-F) 8515771 10.1016/0166-6851(93)90002-f

[10] Burns JM Jr Majarian WR Young JF Daly TM Long CA 1989 A protective monoclonal antibody recognizes an epitope in the carboxyl-terminal cysteine-rich domain in the precursor of the major merozoite surface antigen of the rodent malarial parasite, *Plasmodium yoelii*. J. Immunol. 143, 2670–2676. 2477452

[11] Spencer Valero LM Ogun SA Fleck SL Ling IT Scott-Finnigan TJ Blackman MJ Holder AA 1998 Passive immunization with antibodies against three distinct epitopes on *Plasmodium yoelii* merozoite surface protein 1 suppresses parasitemia. Infect. Immun. 66, 3925–3930. 9673281 10.1128/iai.66.8.3925-3930.1998 PMC108453

[12] Babon JJ Morgan WD Kelly G Eccleston JF Feeney J Holder AA 2007 Structural studies on *Plasmodium vivax* merozoite surface protein-1. Mol. Biochem. Parasitol. 153, 31–40. (doi:10.1016/j.molbiopara.2007.01.015) 17343930 10.1016/j.molbiopara.2007.01.015

[13] Chitarra V Holm I Bentley GA Petres S Longacre S 1999 The crystal structure of C-terminal merozoite surface protein 1 at 1.8 Å resolution, a highly protective malaria vaccine candidate. Mol. Cell 3, 457–464. (doi:10.1016/S1097-2765(00)80473-6) 10230398 10.1016/s1097-2765(00)80473-6

[14] Garman SC Simcoke WN Stowers AW Garboczi DN 2003 Structure of the C-terminal domains of merozoite surface protein-1 from *Plasmodium knowlesi* reveals a novel histidine binding site. J. Biol. Chem. 278, 7264–7269. (doi:10.1074/jbc.M210716200) 12493733 10.1074/jbc.M210716200

[15] Morgan WD Birdsall B Frenkiel TA Gradwell MG Burghaus PA Syed SE Uthaipibull C Holder AA Feeney J 1999 Solution structure of an EGF module pair from the *Plasmodium falciparum* merozoite surface protein 1. J. Mol. Biol. 289, 113–122. (doi:10.1006/jmbi.1999.2753) 10339410 10.1006/jmbi.1999.2753

[16] Morgan WD Frenkiel TA Lock MJ Grainger M Holder AA 2005 Precise epitope mapping of malaria parasite inhibitory antibodies by TROSY NMR cross-saturation. Biochemistry 44, 518–523. (doi:10.1021/bi0482957) 15641776 10.1021/bi0482957

[17] Morgan WD Lock MJ Frenkiel TA Grainger M Holder AA 2004 Malaria parasite-inhibitory antibody epitopes on *Plasmodium falciparum* merozoite surface protein-1(19) mapped by TROSY NMR. Mol. Biochem. Parasitol. 138, 29–36. (doi:10.1016/j.molbiopara.2004.06.014) 15500913 10.1016/j.molbiopara.2004.06.014

[18] Pizarro JC Chitarra V Verger D Holm I Petres S Dartevelle S Nato F Longacre S Bentley GA 2003 Crystal structure of a Fab complex formed with PfMSP1–19, the C-terminal fragment of merozoite surface protein 1 from *Plasmodium falciparum*: a malaria vaccine candidate. J. Mol. Biol. 328, 1091–1103. (doi:10.1016/S0022-2836(03)00376-0) 12729744 10.1016/s0022-2836(03)00376-0

[19] Uthaipibull C 2001 Inhibitory and blocking monoclonal antibody epitopes on merozoite surface protein 1 of the malaria parasite *Plasmodium falciparum*. J. Mol. Biol. 307, 1381–1394. (doi:10.1006/jmbi.2001.4574) 11292349 10.1006/jmbi.2001.4574

[20] Guevara Patino JA Holder AA McBride JS Blackman MJ 1997 Antibodies that inhibit malaria merozoite surface protein-1 processing and erythrocyte invasion are blocked by naturally acquired human antibodies. J. Exp. Med. 186, 1689–1699. (doi:10.1084/jem.186.10.1689) 9362529 10.1084/jem.186.10.1689 PMC2199131

[21] Holder AA Guevara Patino JA Uthaipibull C Syed SE Ling IT Scott-Finnigan T Blackman MJ 1999 Merozoite surface protein 1, immune evasion, and vaccines against asexual blood stage malaria. Parassitologia 41, 409–414. 10697894

[22] Ogutu BR 2009 Blood stage malaria vaccine eliciting high antigen-specific antibody concentrations confers no protection to young children in western Kenya. PLoS One 4, e4708 (doi:10.1371/journal.pone.0004708) 19262754 10.1371/journal.pone.0004708 PMC2650803

[23] Pantophlet R Burton DR 2003 Immunofocusing: antigen engineering to promote the induction of HIV-neutralizing antibodies. Trends Mol. Med. 9, 468–473. (doi:10.1016/j.molmed.2003.09.001) 14604823 10.1016/j.molmed.2003.09.001

[24] Hensmann M Li C Moss C Lindo V Greer F Watts C Ogun SA Holder AA Langhorne J 2004 Disulfide bonds in merozoite surface protein 1 of the malaria parasite impede efficient antigen processing and affect the *in vivo* antibody response. Eur. J. Immunol. 34, 639–648. (doi:10.1002/eji.200324514) 28748538 10.1002/eji.200490004

[25] Piotto M Saudek V Sklenar V 1992 Gradient-tailored excitation for single-quantum NMR spectroscopy of aqueous solutions. J. Biomol. NMR 2, 661–665. (doi:10.1007/BF02192855) 1490109 10.1007/BF02192855

[26] Delaglio F Grzesiek S Vuister GW Zhu G Pfeifer J Bax A 1995 NMRPipe: a multidimensional spectral processing system based on UNIX pipes. J. Biomol. NMR 6, 277–293. (doi:10.1007/BF00197809) 8520220 10.1007/BF00197809

[27] Bartels C Xia TH Billeter M Guntert P Wuthrich K 1995 The program XEASY for computer-supported NMR spectral analysis of biological macromolecules. J. Biomol. NMR 6, 1–10. (doi:10.1007/BF00417486). 22911575 10.1007/BF00417486

[28] Cornilescu G Delaglio F Bax A 1999 Protein backbone angle restraints from searching a database for chemical shift and sequence homology. J. Biomol. NMR 13, 289–302. (doi:10.1023/A:1008392405740) 10212987 10.1023/a:1008392405740

[29] Linge JP Nilges M 1999 Influence of non-bonded parameters on the quality of NMR structures: a new force field for NMR structure calculation. J. Biomol. NMR 13, 51–59. (doi:10.1023/A:1008365802830) 10905826 10.1023/a:1008365802830

[30] Linge JP O'Donoghue SI Nilges M 2001 Automated assignment of ambiguous nuclear Overhauser effects with ARIA. Methods Enzymol. 339, 71–90. (doi:10.1016/S0076-6879(01)39310-2) 11462826 10.1016/s0076-6879(01)39310-2

[31] Nilges M 1995 Calculation of protein structures with ambiguous distance restraints. Automated assignment of ambiguous NOE crosspeaks and disulphide connectivities. J. Mol. Biol. 245, 645–660. (doi:10.1006/jmbi.1994.0053) 7844833 10.1006/jmbi.1994.0053

[32] Nilges M O’ Donoghue SI 1998 Ambiguous NOEs and automated NOE assignment. Prog. Nucl. Magn. Reson. Spectrosc. 32, 107–139. (doi:10.1016/S0079-6565(97)00025-3)

[33] Koradi R Billeter M Wuthrich K 1996 MOLMOL: a program for display and analysis of macromolecular structures. J. Mol. Graph. 14, 51–55. (doi:10.1016/0263-7855(96)00009-4) 8744573 10.1016/0263-7855(96)00009-4

[34] Dayringer HE Tramontano A Sprang SR Fletterick RJ 1986 Interactive program for visualization and modelling of proteins, nucleic acids and small molecules. J. Mol. Graph. 4, 82–87. (doi:10.1016/0263-7855(86)80002-9)

[35] Laskowski RA Rullmannn JA MacArthur MW Kaptein R Thornton JM 1996 AQUA and PROCHECK-NMR: programs for checking the quality of protein structures solved by NMR. J. Biomol. NMR 8, 477–486. (doi:10.1007/BF00228148) 9008363 10.1007/BF00228148

[36] Doreleijers JF Rullmann JA Kaptein R 1998 Quality assessment of NMR structures: a statistical survey. J. Mol. Biol. 281, 149–164. (doi:10.1006/jmbi.1998.1808) 9680482 10.1006/jmbi.1998.1808

[37] Moss DK Remarque EJ Faber BW Cavanagh DR Arnot DE Thomas AW Holder AA 2012 *Plasmodium falciparum* 19-kilodalton merozoite surface protein 1 (MSP1)-specific antibodies that interfere with parasite growth *in vitro* can inhibit MSP1 processing, merozoite invasion, and intracellular parasite development. Infect. Immun. 80, 1280–1287. (doi:10.1128/IAI.05887-11) 22202121 10.1128/IAI.05887-11 PMC3294643

[38] Arnot DE Cavanagh DR Remarque EJ Creasey AM Sowa MP Morgan WD Holder AA Longacre S Thomas AW 2008 Comparative testing of six antigen-based malaria vaccine candidates directed toward merozoite-stage *Plasmodium falciparum*. Clin. Vaccine Immunol. 15, 1345–1355. (doi:10.1128/CVI.00172-08) 18550731 10.1128/CVI.00172-08 PMC2546674

[39] Draper SJ Moore AC Goodman AL Long CA Holder AA Gilbert SC Hill F Hill AV 2008 Effective induction of high-titer antibodies by viral vector vaccines. Nat. Med. 14, 819–821. (doi:10.1038/nm.1850) 18660818 10.1038/nm.1850 PMC4822545

[40] Faber BW Remarque EJ Morgan WD Kocken CH Holder AA Thomas AW 2007 Malaria vaccine-related benefits of a single protein comprising *Plasmodium falciparum* apical membrane antigen 1 domains I and II fused to a modified form of the 19-kilodalton C-terminal fragment of merozoite surface protein 1. Infect. Immun. 75, 5947–5955. (doi:10.1128/IAI.01804-06) 17938224 10.1128/IAI.01804-06 PMC2168333

[41] Drew DR O'Donnell RA Smith BJ Crabb BS 2004 A common cross-species function for the double epidermal growth factor-like modules of the highly divergent plasmodium surface proteins MSP-1 and MSP-8. J. Biol. Chem. 279, 20 147–20 153. (doi:10.1074/jbc.M401114200) 10.1074/jbc.M401114200 14976193

[42] Okafor CM 2009 Cellular responses to modified *Plasmodium falciparum* MSP1_19_ antigens in individuals previously exposed to natural malaria infection. Malar. J. 8, 263 (doi:10.1186/1475-2875-8-263) 19930613 10.1186/1475-2875-8-263 PMC2785830

[43] de Koning-Ward TF O'Donnell RA Drew DR Thomson R Speed TP Crabb BS 2003 A new rodent model to assess blood stage immunity to the *Plasmodium falciparum* antigen merozoite surface protein 1_19_ reveals a protective role for invasion inhibitory antibodies. J. Exp. Med. 198, 869–875. (doi:10.1084/jem.20030085) 12963693 10.1084/jem.20030085 PMC2194199

[44] Murhandarwati EE Wang L Black CG Nhan DH Richie TL Coppel RL 2009 Inhibitory antibodies specific for the 19-kilodalton fragment of merozoite surface protein 1 do not correlate with delayed appearance of infection with *Plasmodium falciparum* in semi-immune individuals in Vietnam. Infect. Immun. 77, 4510–4517. (doi:10.1128/IAI.00360-09) 19620342 10.1128/IAI.00360-09 PMC2747923

[45] McIntosh RS 2007 The importance of human FcgammaRI in mediating protection to malaria. PLoS Pathog. 3, e72 (doi:10.1371/journal.ppat.0030072) 17511516 10.1371/journal.ppat.0030072 PMC1868954

[46] Lazarou M 2009 Inhibition of erythrocyte invasion and *Plasmodium falciparum* merozoite surface protein 1 processing by human immunoglobulin G1 (IgG1) and IgG3 antibodies. Infect. Immun. 77, 5659–5667. (doi:10.1128/IAI.00167-09) 19805526 10.1128/IAI.00167-09 PMC2786472

[47] Coley AM 2006 The most polymorphic residue on *Plasmodium falciparum* apical membrane antigen 1 determines binding of an invasion-inhibitory antibody. Infect. Immun. 74, 2628–2636. (doi:10.1128/IAI.74.5.2628-2636.2006) 16622199 10.1128/IAI.74.5.2628-2636.2006 PMC1459722

