# Supplementary material for: The structure of Plasmodium yoelii merozoite surface protein 119, antibody specificity and implications for malaria vaccine design
Source: Open Biol. 2014 Jan 8;4(1):130091. doi: 10.1098/rsob.130091 (PMC3909271; doi:10.1098/rsob.130091)
Supplement: Py MSP1 supplementary table and figures [file rsob130091supp1.pdf]

Table S1: Structural data

ARIA2.2 Structure calculations		
	Wild type	E28K
total restraints used	3391	2927
	(% unambiguous)	(% unambiguous)
^{15}N NOESY-HSQC	729 (64)	476 (71)
^{13}C NOESY-HSQC aliphatic	2083 (73)	1904 (75)
^{13}C NOESY-HSQC aromatic	71 (68)	63 (81)
^{1}H NOESY in D_2O	508 (58)	485 (59)
total violations	2	6
RMSD –superimposed family		
backbone	0.45 (10)	0.54 (10)
heavy atoms	1.0 (10)	1.1 (10)
Ramachandran	%	%
most favoured regions	62.1	67.6
allowed regions	31.1	29.3
generously allowed regions	5.4	2.2
disallowed regions	1.4	1.0

Figure S1. $^{1}H\{^{15}N\}$-HSQC spectrum for wild type *P. yoelii* $MSP1_{19}$ with resonance assignments indicated.

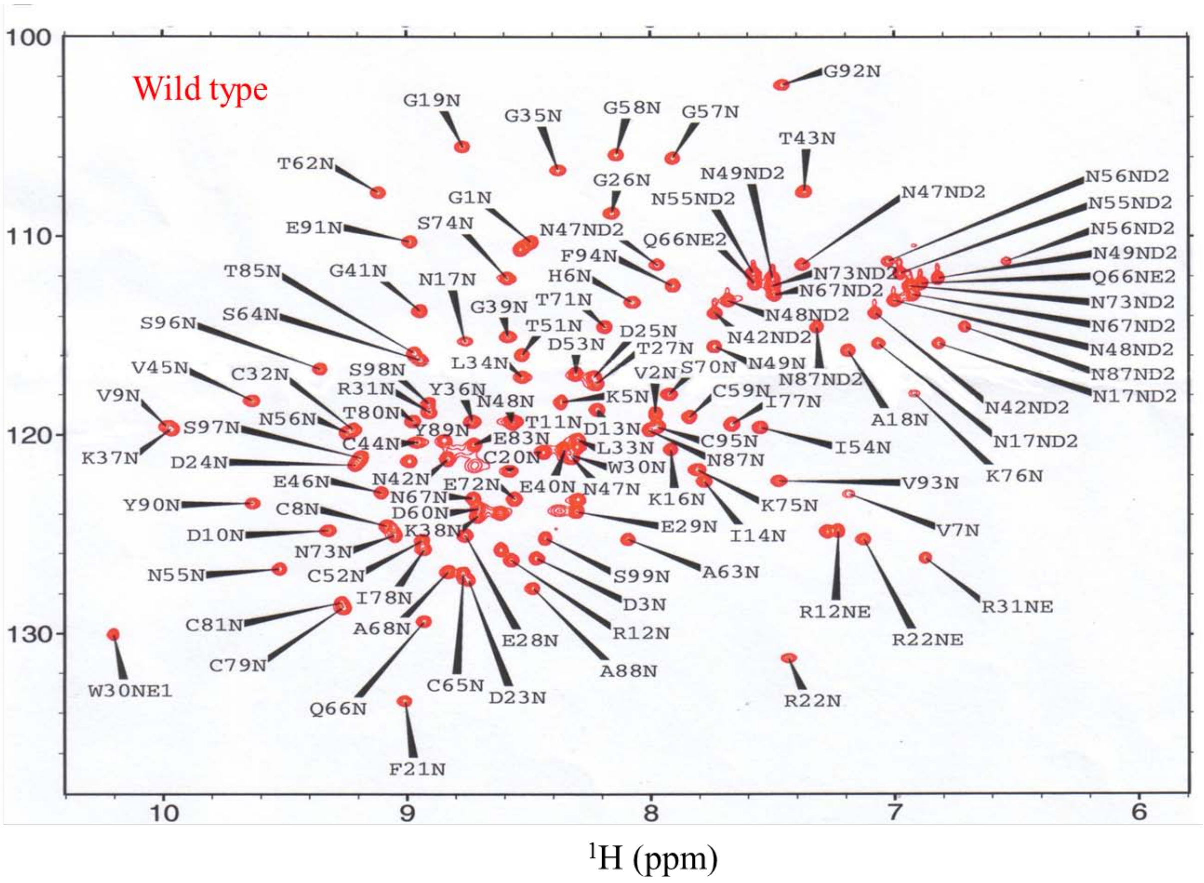

Figure S2. The effects of single amino acid substitutions on the structure of $MSP1_{19}$. (a) Comparison of the $^{1}H\{^{15}N\}$-HSQC spectra for wild type (red) and K16E (light blue) $MSP1_{19}$. (b) Comparison of the $^{1}H\{^{15}N\}$-HSQC spectra for wild type (red) and N17H (purple) $MSP1_{19}$.

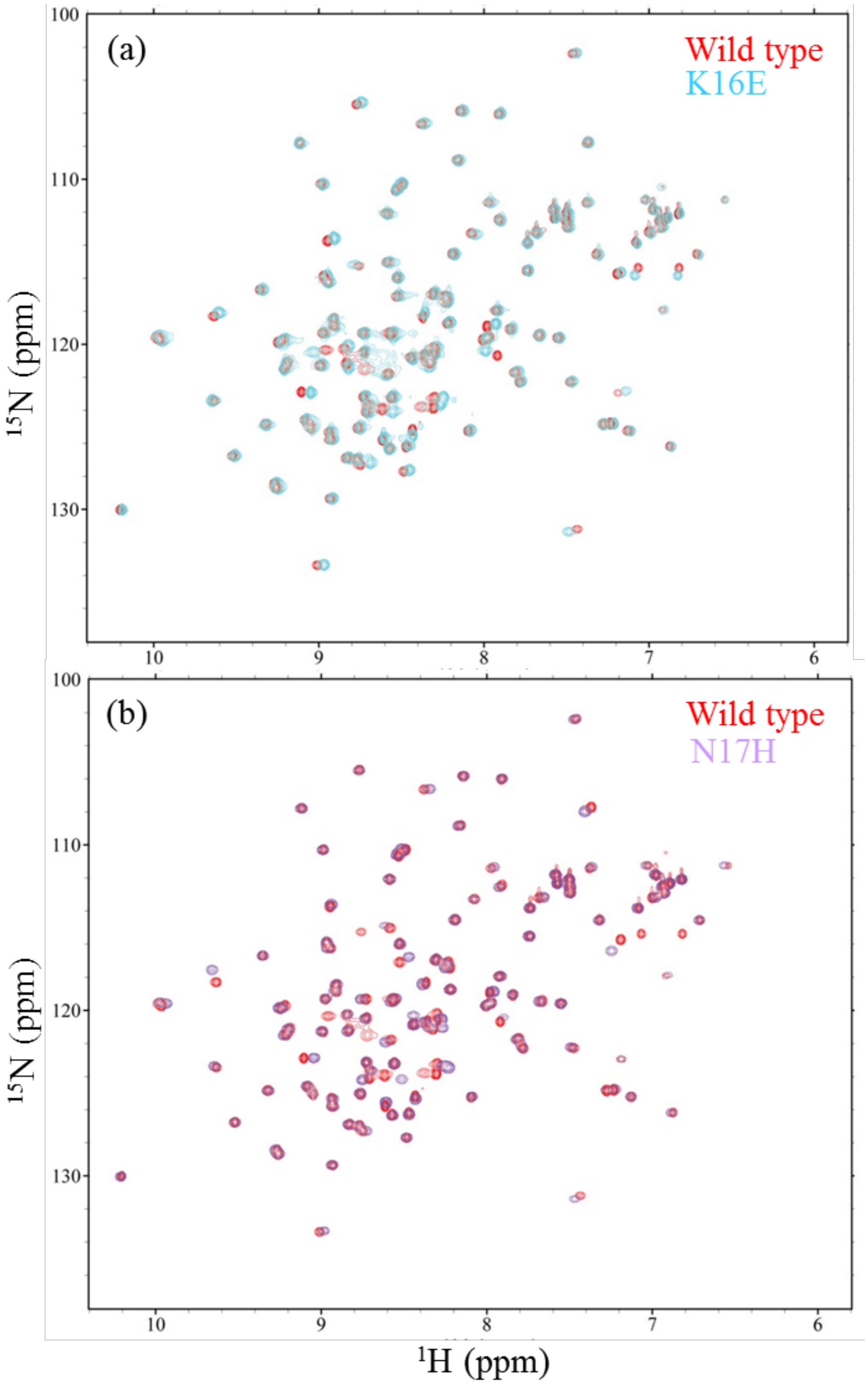

Figure S3. D_2O exchange rates for individual residues in wild type (red) and E28K variant (blue) $MSP1_{19.}$ D_2O exchange rates were calculated from a D_2O exchange time course experiment in which the proteins were dissolved in D_2O and ^{15}N-HSQC were acquired every 5 minutes for 2 – 3 hours at 25 ^{o}C. The NHs that were exchanging with the D_2O with a half time of less than 5 minutes were given an arbitrary value of 1 and the NHs that were exchanging with the D_2O with a half time of more than 3000 minutes were given an arbitrary value of 3000. Proline residues were assigned a value of 1 as they have no NH to exchange.

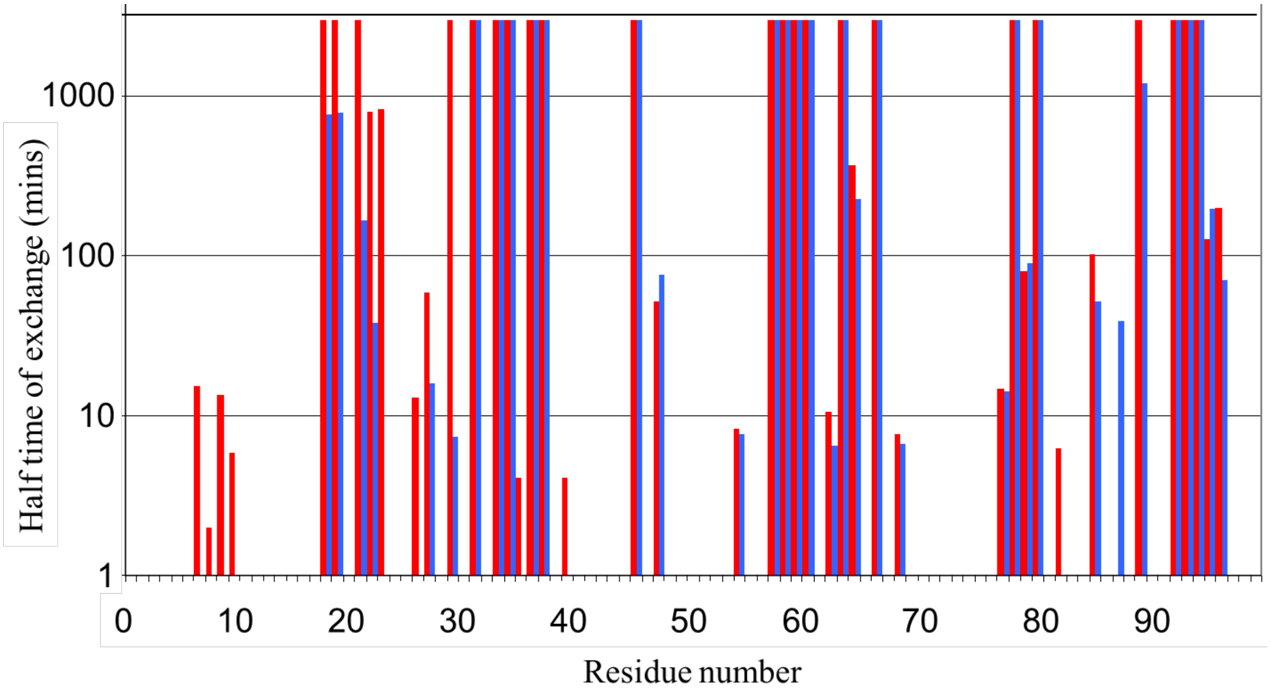

Figure S4. Comparison of the 3D structures of WT and E28K $MSP1_{19}$ variant. (a) Calculated 20 lowest energy NMR structures for the WT protein (first and second EGF-like domains shown in red and orange, respectively) and the variant protein (first and second EGF-like domains shown in blue and cyan, respectively).

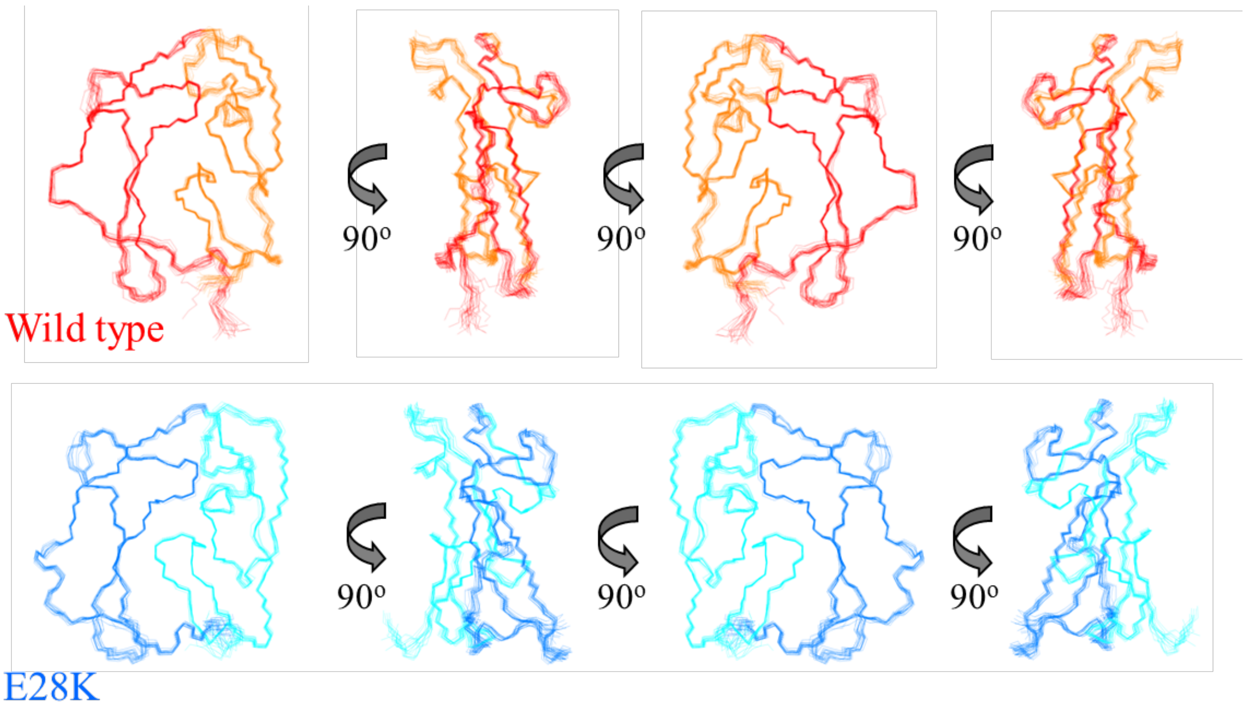

Figure S5. Survival after *P. yoelii* YM parasite challenge following immunisation with wild type and variant $MSP1_{19}$ antigens in a mouse model. Groups of mice (n = 12) immunized with the R12L, K16E and N17H variants exhibited comparable survival rates to that observed with WT $MSP1_{19}$ ($p>0.3$). The control group immunised with GST and mice immunised with E28K variant antigen exhibited significantly lower survival rates ($p<0.0001$). Results are shown as Kaplan-Meier survival curves, and the differences in survival were calculated by log rank analysis.

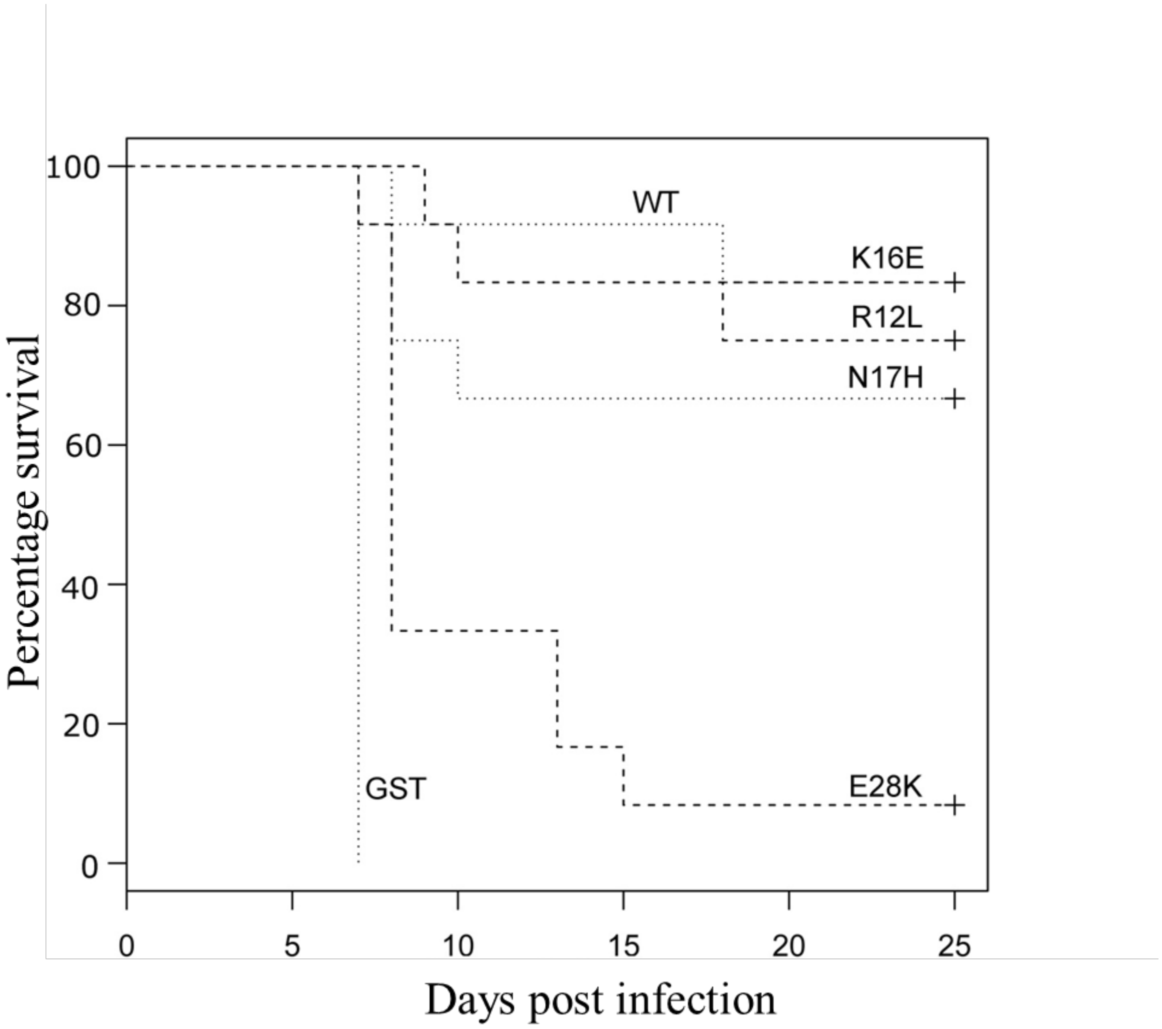

Figure S6. An alignment of $MSP1_{19}$ amino acid sequences from *Plasmodium falciparum* (Pf), *P. yoelii* (Py), *P. berghei* (Pb), *P. chabaudi* (Pc), *P. vivax* (Pv), *P. knowlesi* (Pk) and *P. cynomolgi* (Pc). The two EGF-like domains are indicated. The conserved cysteine residues of the *P. falciparum* sequence and the corresponding residues of the other sequences, including the Ile/Val/Thr and Trp replacements, are indicated in yellow. Other residues conserved across all species are highlighted in grey and the region perturbed in the E28K variant of *P. yoelii* $MSP1_{19}$ is highlighted in black. The same color-coding is used for the 3-dimensional structures of *P. yoelii* WT and E28K, *P. cynomolgi* (1B9W) and *P. falciparum* (1CEJ) $MSP1_{19}$.

```
                10         20         30         40         50         60         70         80         90         100
        .........| .........| .........| .........| .........| .........| .........| .........| .........| .........|
Pf      NIS-QHQCVK K-QCPQNSGC FRHLDEREEC KCLLNYK--Q EGDKCVENPN PTCNENNGGC DADAKCTEED SGSN--GKKI TCECTKPDSY PLFDGIFCSS
Py      GVDPKHVCVD TRDIPKNAGC FRDDNGTEEW RCLLGYKKGE -GNTCVENNN PTCDINNGGC DPTASCQNAE STEN--SKKI ICTCKEPTPN AYYEGVFCSS
Pb      GIDPKHVCIN TRDIPANAGC FRYDNGNEEW RCLLGYKKNN --NTCIEDSN PTCGNNNGGC DPTAGCQTAE NREN--SKKI ICTCKEPTPN AYYDGVFCSS
Pc      GIGSNHVCIS T-STPDNAGC FRYDDGTEEW RCLLGFKKDD DGNRCVADDA PVCNNNNGGC DKNADCREVE NTDRDPSKKI VCTCKEPNPN AYYAGVFCSS
Pv      TMSSEHTCID T-NVPDNAAC YRYLDGMEEW RCLLTFK--E EGGKCVPGSN VTCKDNNGGC APEAECKMTD SN------KI VCKCTKEGSE PLFEGVFCSS
Pk      NMSSAHKCID T-NVPENAAC YRYLDGTEEW RCLLGFK--E VGGKCVPAS- ITCEENNGGC APEAECTMED ------KKEV ECKCTKEGSE PLFEGVFCSS
Pcy     NMSSEHRCID T-NVPENAAC YRYLDGTEEW RCLLYFK--E DAGKCVPAPN MTCKDKNGGC APEAECKMND ------KNEI VCKCTKEGSE PLFEGVFCSS
              ← First Epidermal Growth Factor-like domain →       ←     Second Epidermal Growth Factor-like domain     →
```

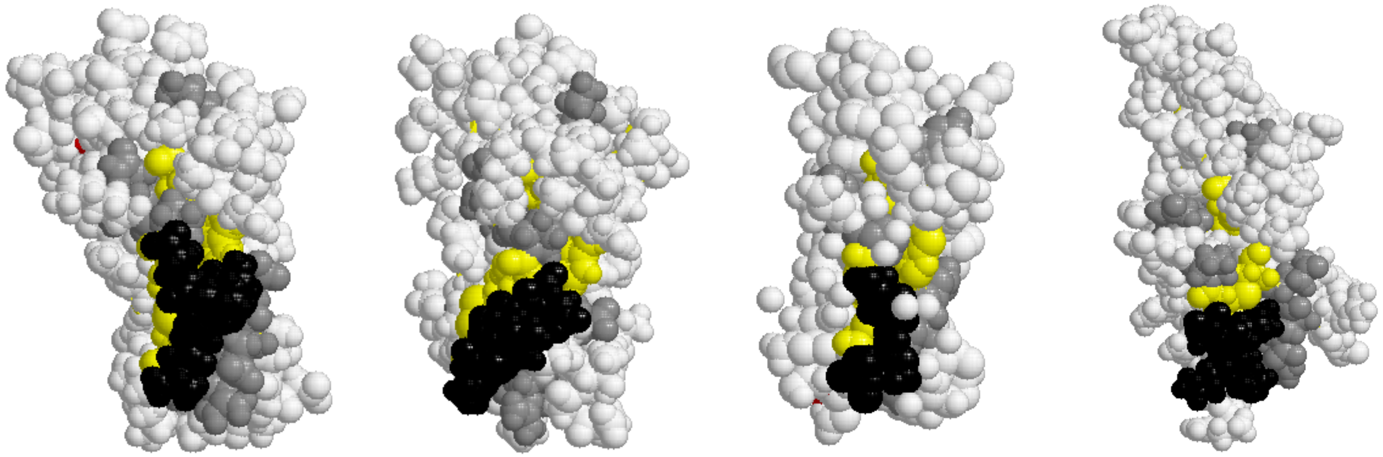

P. yoelli WT   *P. yoelli* E28K   *P. cynomolgi*   *P. falciparum*
